# P-1088. Safety Study of Rifaquizinone in Dogs Following Intra-articular Administration

**DOI:** 10.1093/ofid/ofae631.1276

**Published:** 2025-01-29

**Authors:** Huan Wang, Qin Cheng, Weijian Ke, Zhenkun Ma

**Affiliations:** TenNor Therapeutics (Suzhou) Ltd, Suzhou, Jiangsu, China (People's Republic); TenNor Therapeutics (Suzhou) Ltd, Suzhou, Jiangsu, China (People's Republic); WuXi AppTec (Suzhou) CO., Ltd., Suzhou, Jiangsu, China; TenNor Therapeutics, Suzhou Industrial Park, Jiangsu, China (People's Republic)

## Abstract

**Background:**

Rifaquizinone (RFQ, TNP-2092) is a novel multitargeting drug conjugate in development for the treatment of prosthetic joint infections (PJIs). RFQ exerts its antibacterial activity by inhibiting RNA polymerase, DNA gyrase, and topoisomerase IV. This study sought to investigate the safety/tolerability of RFQ following intra-articular (IA) administration in Beagle dogs.

**Figure:**
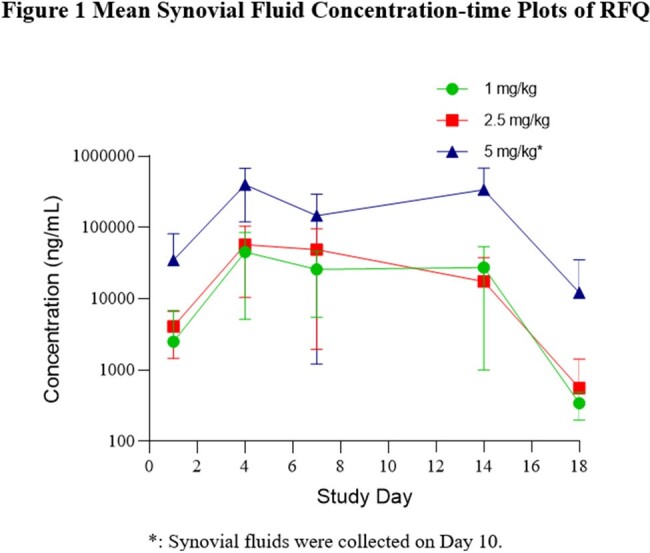

**Methods:**

Forty Beagle dogs (20/sex) were randomly assigned to 4 groups (5/sex/group) to receive 0, 1, 2.5, and 5 mg/kg/day RFQ in 5% (w/v) glucose once daily for 14 days by IA administration into the right knee joint. All the dogs had a 14-day recovery period to assess the reversibility, persistence, or delayed occurrence of adverse effects. Blood and synovial fluid samples were collected and concentrations of RFQ in each matrix were determined by liquid chromatography tandem mass spectrometry.

**Results:**

No test article-related adverse effects in body weight, food consumption, temperature, clinical pathology, or organ weight were observed in any of the study groups. In 2.5 and 5 mg/kg/day groups, lameness, swelling and ulceration of right knee joint during treatment were observed. The 5 mg/kg group dosing was terminated prematurely due to marked arthritis on right knee joint. The no observed adverse effect level (NOAEL) was 1 mg/kg/day. At this dose level the steady state of RFQ concentrations in synovial fluid was achieved in 4 days and maintained until Day 14 (Figure 1). The mean RFQ concentrations on Day 14 in synovial fluids for male and female dogs were 35,900 and 19,200 ng/mL, respectively, as compared to the plasma C_max_ 411 and 303 ng/mL for male and female dogs, respectively. Plasma toxicokinetics (TK) analysis indicated no apparent sex differences in systemic exposure at any dose level.

**Conclusion:**

The NOAEL of RFQ in dogs was 1 mg/kg/day via IA administration. The synovial fluid concentration at this dose level exceeded minimum biofilm bactericidal concentration inhibit 90% bacterial growth (MBBC_90_) levels for both S. aures and S. epidermidis. IA administration of RFQ is a promising option for the treatment of PJIs.

**Disclosures:**

**Huan Wang, PhD**, TenNor Therapeutics (Suzhou) Ltd: Employee **Qin Cheng, Master**, TenNor Therapeutics (Suzhou) Ltd: Employee **Weijian Ke, Master**, TenNor Therapeutics (Suzhou) Ltd: Investigator **Zhenkun Ma, PhD**, TenNor Therapeutics (Suzhou) Ltd: Employee

